# Quantifying the Improvement in Dielectric Properties of BaSrTiO_3_-Based Ceramics by Adding MgO

**DOI:** 10.3390/ma15082875

**Published:** 2022-04-14

**Authors:** Kun Dai, Ruina Ma, Xing Wang, Zhaoyang Zheng, Yongzhe Fan, Xue Zhao, An Du, Xiaoming Cao

**Affiliations:** Key Lab for New Type of Functional Materials in Hebei Province, Tianjin Key Lab Material Laminating Fabrication and Interface, School of Material Science and Engineering, Hebei University of Technology, Tianjin 300132, China; dk1037667756@foxmail.com (K.D.); maryna@126.com (R.M.); wangxinggr@163.com (X.W.); zzy983608892@163.com (Z.Z.); fyz@hebut.edu.cn (Y.F.); zhaoxue@hebut.edu.cn (X.Z.); gd_sam@galvanize.com.cn (X.C.)

**Keywords:** MgO, BaTiO_3_, dielectric property, microstructure

## Abstract

Barium titanate (BaTiO_3_, BT) is the main raw material of multilayer ceramic capacitors. As thinner layers of dielectric elements require smaller BT grain diameters, BT-MgO composites have been widely studied owing to the plasticity of MgO and its inhibition of grain growth. However, further improvements of the dielectric properties of the BT-MgO system are still urgently needed. Herein, composite ceramics of Ba_0.7_Sr_0.3_Ti_0.9925_Tm_0.01_O_3_ (BST)-*x* mol% MgO (*x* = 1, 2, 3, 4, 5) were prepared. The dielectric constant of BST-1 mol% MgO at room temperature was approximately 3800, which was 1/3 times higher than that of BT-MgO composite ceramics. The dielectric loss was less than 0.004 and 2/3 that of BT-MgO composite ceramics. The Curie temperature of BST doped with MgO was below 0 °C. The anomalous increase in dielectric constant was caused by the co-doping of Sr and Tm with BT, while the reduced dielectric loss was due to the uniform dispersion of MgO at grain boundaries, which hinders grain growth. The Curie temperature shift was mainly due to accumulated oxygen vacancies. Thus, this work provides new solutions to further improve the dielectric properties of the BT-MgO system, including changing the doping elements and adjusting the doping ratio.

## 1. Introduction

Barium titanate (BaTiO_3_, BT) is still the most widely used functional ferroelectric material, especially as a dielectric in multilayer capacitors due to its excellent electrical properties (high piezoelectric coefficient, high dielectric constant, and positive temperature coefficient effect) [1,2,3,4,5,6]. Therefore, BT has been continuously studied, and various properties have been improved through the chemical modification of its surface. Several single-phase dielectric, ferroelectric, and pyroelectric materials cannot be directly used and require modification due to the challenges in obtaining all essential properties simultaneously. For example, BaSrTiO_3_ (BST)-based ceramics [7,8] have a high dielectric constant but high dielectric loss and low temperature stability; thus, they need to be doped or compounded to reduce dielectric loss. Currently, the synthesis of BT-metal ceramic composites has become an effective method for improving dielectric properties. Due to the plasticity of the metal, the internal stress of the BT-based materials can be relaxed effectively during sintering, and the properties of the matrix can be improved [9,10,11,12,13,14,15].

In recent years, several chemical additives such as MgO and Al_2_O_3_ have been studied. It was found that composite ceramics based on BT, such as BT-MgO [16], BT-Al_2_O_3_ [17,18,19], and BT-BiYO_3_ [20,21], have a high dielectric constant and high breakdown strength; thus, they have been considered as promising energy storage materials. At the same time, MgO has been used to prepare various functional materials due to its high breakdown strength, which can inhibit grain growth at grain boundaries [15]. Following high-temperature sintering, one of the three consequences for Mg^2+^ in BT may occur: some Mg^2+^ can replace Ti^4+^ in the BT Lattice, some Mg^2+^ can react with the BT matrix to form MgTiO_3_, and some Mg^2+^ can still form MgO at the grain boundary owing to the low diffusion rate of Mg^2+^ [22]. The influence of MgO on the dielectric properties of BT-based ceramics has been studied. Park et al. [23] prepared MgO-coated BT particles with a dielectric constant of approximately 2700 at room temperature (≈25 °C) by a homogeneous precipitation method. The results revealed that the dielectric properties of the particles are closely related to the distribution of MgO on the BaTiO_3_ matrix. Li et al. [24] prepared Sc_2_O_3_ and MgO co-doped BT-based ceramics, and measured the dielectric properties of the ceramics. The simultaneous addition of Sc^3+^ and Mg^2+^ resulted in the formation of a core-shell structure. The best dielectric properties of the material were obtained upon the addition of 0.45% mol Sc_2_O_3_ and 1% mol MgO. Under these conditions, the material had a dielectric constant of 1744 with a dielectric loss of 0.58% at room temperature. Currently, researchers are aware of the need to improve the dielectric properties of capacitors, and the real challenge is improving the dielectric properties of the BT-MgO system. In general, the excess MgO in the BT-MgO matrix tends to aggregate at grain boundaries, which inhibits grain growth and reduces the dielectric constant of composite ceramics. Therefore, the dielectric properties of MgO can be improved only by enhancing the matrix.

Our previous studies have revealed the large dielectric constant of the BST matrix. The dielectric constant of the BT matrix was enhanced by co-doping with Sr and Tm, and the dielectric constant at room temperature reached a value of 760,000 [15,25]. Therefore, it is expected that the dielectric properties of the BT-MgO system can be further improved by the application of a matrix with a high dielectric constant.

The aim of this work is to improve the dielectric properties of the BT-MgO system. In this work, BST-*x* mol% MgO (*x* = 1, 2, 3, 4, 5) composite ceramics were prepared, and the effects of varying the MgO content on the phase structure, dielectric properties, and microstructure were studied. 

## 2. Materials and Methods

All medicines were purchased from Sinopharm, Beijing, China. Ba_0.7_Sr_0.3_Ti_0.9925_Tm_0.01_O_3_ ceramic powders were prepared by sol-gel method [25]. In a beaker, appropriate volumes of deionized water and anhydrous ethanol were added to BST powder (x g), and the beaker was then placed in an ultrasonic bath for 45 min to fully disperse the powder. The suspension was initially magnetically stirred at room temperature, and then at 55 °C for 35 min. Then, an aqueous magnesium chloride hexahydrate solution was slowly added, followed by the addition of a urea solution. The pH was then recorded. The suspension was then stirred at 55 °C for 2 h. The mixture was left still in a water bath at 90 °C for 12 h and finally washed and dried. The dried samples were sintered at 700 °C for 3 h, and the sintered samples were finely pulverized. The sample was then accurately weighed, poured into a clean mold, and then pressed under a pressure tester. The speed of the press block was adjusted to 0.1 kN s^−1^ under a pressure of 25 kN, the pressure was held for 5 min, and the pressed sample was obtained. The powder was sintered at 1320 °C for 2 h. The sintered ceramic block was coated with silver on both sides and then placed at 550 °C for 20 min to complete the electrode sintering.

The X-ray diffraction (XRD) patterns of BST and BST-MgO ceramics were recorded on a Bruker D8 Advance X-ray diffractometer (Karlsruhe, Germany) equipped with CuKα radiation (λ = 1.54059 Å) operating at 25 °C over an angular range of 10° to 90° at a rate of 12° min^−1^. The local structures of the samples were detected by an Invia Raman microscope spectrometer (InVia Reflex, Renishaw, UK). The morphologies of the particles were characterized by field-emission transmission electron microscopy (FE-TEM; Tecnai G2 Spirit TWIN, FEI, Hillsboro, OR, USA), energy-dispersive X-ray spectroscopy (EDS), and field-mission scanning electron microscopy (FE-SEM; Nova Nano SEM450, FEI, Hillsboro, OR, USA). The dielectric-frequency curves were recorded using an LCR meter (LCR-800, GW Instek, Taipei, Taiwan) in the frequency range of 0.12–200 kHz at room temperature, and the dielectric-temperature curves were recorded using an LCR meter (Agilent E4980A, Santa Clara, CA, USA) at a frequency of 1 kHz.

## 3. Results and Discussion

### 3.1. XRD Analysis of BST-MgO Ceramic Powder

**Figure 1:**
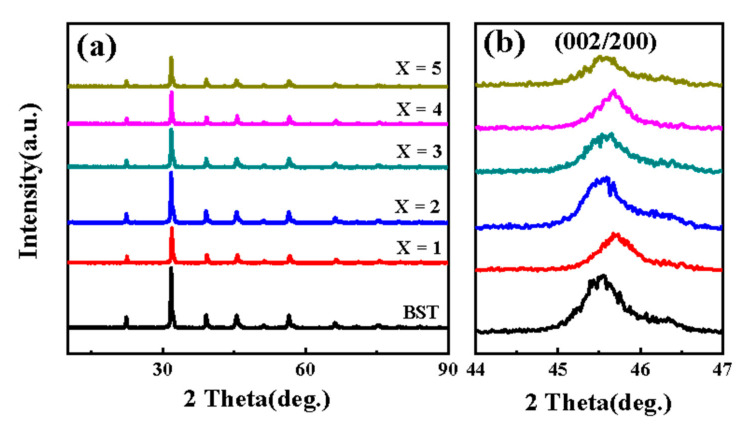
(**a**) XRD patterns of BST and BST-*x* mol% MgO (*x* = 1, 2, 3, 4, 5) powders. (**b**) Magnification of the patterns shown in (**a**) at the range of 44° to 47°.

The XRD patterns of BST and BST-*x* mol% MgO (*x* = 1, 2, 3, 4, 5) ceramic powder are shown in Figure 1. The BST with *x* mol% MgO ceramic powder did not generate a heterogenous phase after MgO doping, and all ceramic powders presented a single perovskite structure. The experimental XRD pattern of the BST ceramic powder agreed with JCPDS card number 89-0274, and that of the BST-MgO ceramic powder agreed with JCPDS card number 44-0093. The ceramic powders of BST and BST with varying *x* mol% of MgO were unimodal with a pseudo cubic structure. The results show that adding a small amount of MgO to the BST-x mol% MgO (*x* = 1, 2, 3, 4, 5) ceramic powder has no obvious effect on the powder crystal structure. Table 1 lists all the lattice parameters of the BST and BST-*x* mol% MgO ceramic powders. As the content of MgO increased, the lattice constant of the ceramic powders increased, the *c*/*a* ratio increased, and the stability of the crystal structure of the pseudo cubic phase was enhanced.

### 3.2. TEM Analysis of BST-MgO Ceramic Powder

**Figure 2:**
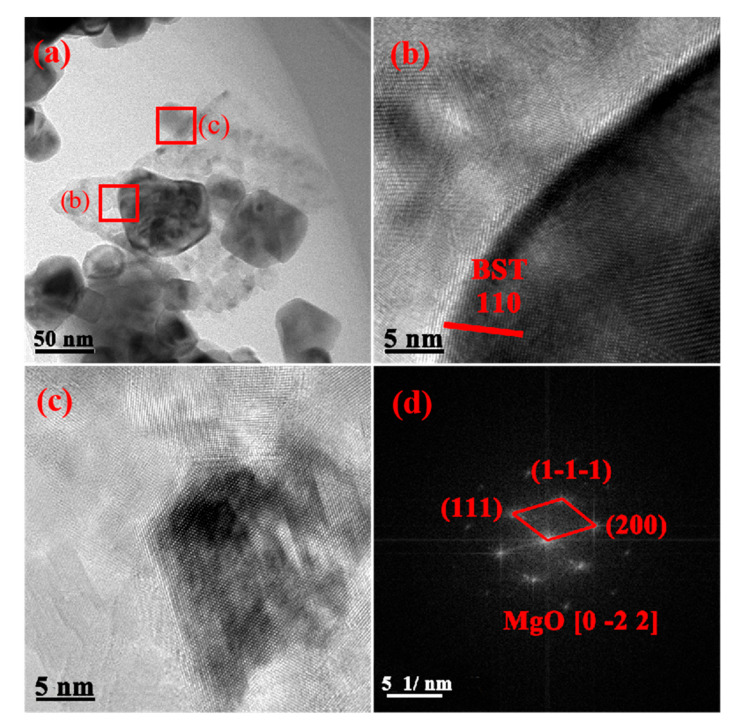
(**a**) TEM image of the BST-2 mol% MgO powder. (**b**) The high-resolution diagram of (**a**). (**c**) The high-resolution diagram of (**a**). (**d**) The Fourier transform figure of (**c**).

Figure. 2 is a transmission morphology diagram of BST-2 mol% MgO ceramic powder. The ceramic particle size was in the range of 40–55 nm (Figure 2a). At the same time, the XRD results of BST-2 mol% MgO ceramic particles were analyzed using MDI Jade to obtain peak widths, and the crystal size was ascertained using Scherrer’s equation. The calculations showed that the particle size of the ceramic particles was approximately 45 nm, which is consistent with the results in the transmission spectrum. The lattice fringe spacing in Figure 2b was 0.372 nm, which corresponds to the (110) plane of BST. Notably, MgO was unevenly distributed around the BST particles. The high-resolution diagram of MgO is shown in Figure 2c; simultaneously, Fourier transform was performed on the area shown in Figure 2c to obtain that which is shown in Figure 2d. The diffraction spots were calibrated, and the phase of MgO was preliminarily determined.

### 3.3. Infrared Spectrum Analysis of BST-MgO Ceramic Powder

**Figure 3:**
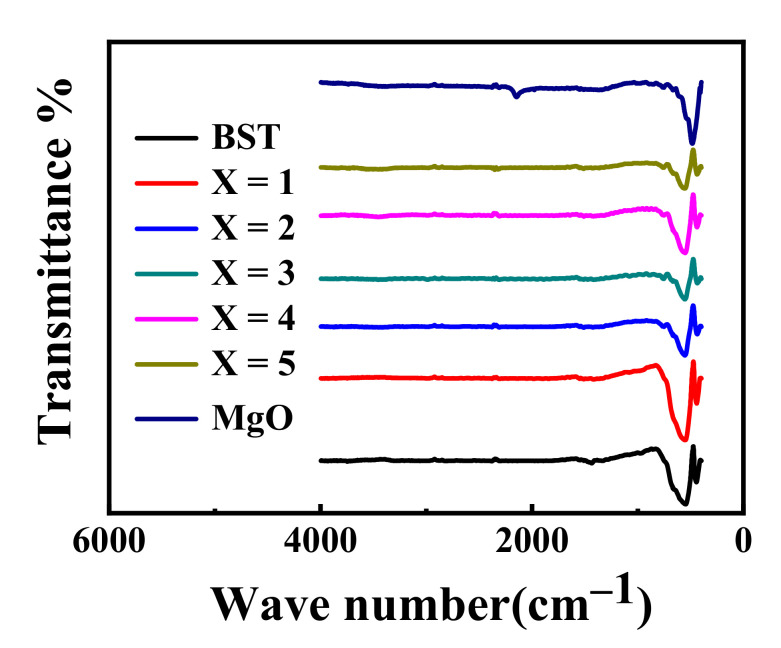
The infrared spectra of BST and BST-*x* mol% MgO (*x* = 1, 2, 3, 4, 5) powders.

Figure 3 shows the infrared spectra of the BST and BST-*x* mol% MgO ceramic powders. The figure reveals that the Ti-O bond absorption peak of the BST structure was displayed at 534 cm^−1^. With the increase in MgO content, the Ti-O bond absorption peaks of BST-x mol% MgO ceramic particles are 534 cm^−1^ (*x* = 1), 532 cm^−1^ (*x* = 2), 532 cm^−1^ (*x* = 3), 529 cm^−1^ (*x* = 4) and 528 cm^−1^ (*x* = 5). Comparing the absorption peaks of the BST and BST-*x* mol% MgO (*x* = 1, 2, 3, 4, 5) ceramic powders in the figure, it was found that the Ti-O bond absorption peak intensity of the BST-1 mol% MgO ceramic powder was the strongest, and the Ti-O bond intensity became weaker with an increase in the MgO content. The peak of the Ti-O bond moved to a lower wavenumber. Since the ionic radii of Mg^2+^ (*r* = 0.072 nm) and Ti^4+^ (*r* = 0.061 nm) are similar, Mg^2+^ replaced Ti^4+^ in the BST crystal lattice. The oxygen octahedron structure of BST was distorted due to the substitution of non-equivalent elements, resulting in the change of the Ti-O bond peak [15]. There was no absorption peak of MgO in the BST-*x* mol% MgO ceramic powders in the figure, which may be due to the low MgO content.

### 3.4. XRD Analysis of BST-MgO Ceramic Block

**Figure 4:**
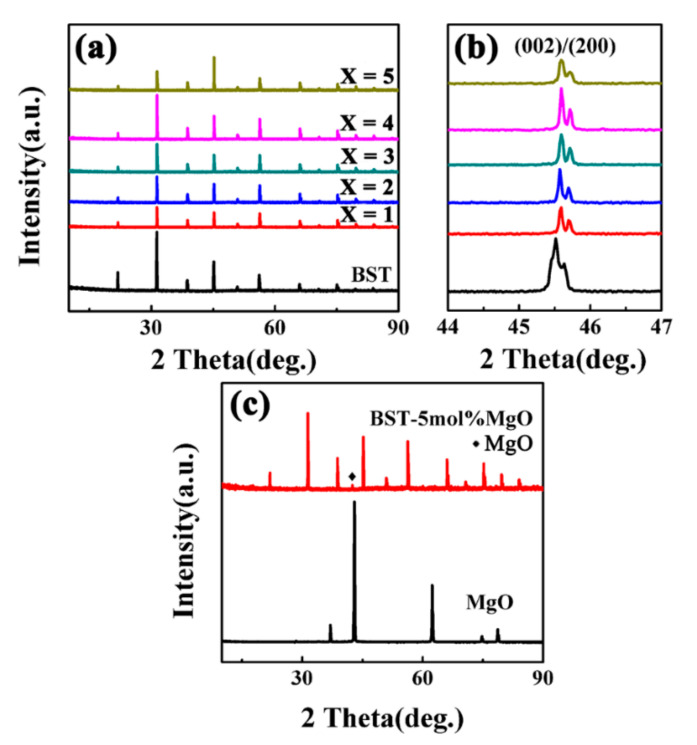
(**a**) XRD patterns of BST and BST-*x* mol% MgO (*x* = 1, 2, 3, 4, 5) ceramics. (**b**) Magnification of the patterns shown in (**a**) at the range of 44° to 47°. (**c**) XRD patterns of BST-5 mol% MgO ceramic and MgO.

Figure 4 shows the XRD patterns of ceramic blocks after sintering. The results revealed that all of the sintered ceramics showed a pure perovskite phase. Figure 4b shows that the (002)/(200) peak of pure BST was a single peak with a pseudo cubic structure. After doping with MgO, the (002)/(200) peak of BST-*x* mol% MgO became bimodal with a tetragonal phase structure. Moreover, the (002)/(200) peak of BST-*x* mol% MgO gradually moved to a higher angle than that of BST. As the ionic radii of Mg^2+^ and Ti^4+^ are similar, Mg^2+^ replaced Ti^4+^, and a small amount of Mg^2+^ might have entered the BST perovskite crystal. This shifts the diffraction peak of BST-x mol% MgO ceramic blocks moved to a higher angle [22]. To further confirm this change, Table 2 provides the lattice parameters of all ceramic blocks. The lattice constant of the ceramic blocks (Table 2) increased because Ti^4+^ was replaced by Mg^2+^.

### 3.5. Raman Analysis of BST-MgO Ceramics

**Figure 5:**
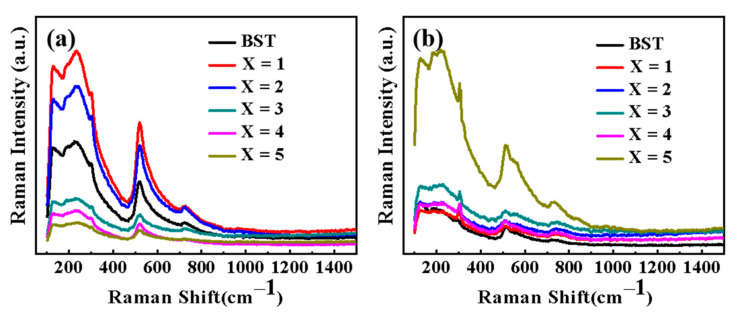
(**a**) Room-temperature Raman spectroscopy results for BST and BST-x mol% MgO (x = 1, 2, 3, 4, 5) powders. (**b**) Room-temperature Raman spectroscopy results for BST and BST-x mol% MgO (x = 1, 2, 3, 4, 5) ceramics.

BST had four peaks at 247 cm^−1^, 301 cm^−1^, 522 cm^−1^, and 729 cm^−1^ (Figure 5). From Figure 5a, it can be found there was no change in the shapes of all Raman peaks, indicating that the crystal structure did not change after the addition of MgO. The characteristic peak of the tetragonal phase is at 301 cm^−1^. It can be seen from the figure that BST and BST-*x* mol% MgO had a small peak at 301 cm^−1^ without a tetragonal feature, which corroborates the XRD results of the ceramic powders. All ceramic powders had a pseudo cubic phase structure. Notably, BST-x mol% MgO ceramic powders revealed sharper peaks at 247 cm^−1^, 522 cm^−1^, and 729 cm^−1^ compared with BST, and a blue shift occurred at 522 cm^−1^ and 729 cm^−1^. As the shift of a Raman peak is related to the movement of atoms in the crystal lattice, the lattice constant of BST-*x* mol% MgO increased compared with that of BST, which eventually resulted in the Raman peak moving to a higher angle. It can be seen from Figure 5b that the Raman peak of BST-*x* mol% MgO was sharp at 301 cm^−1^. This indicates that the BST-*x* mol% MgO (*x* = 1, 2, 3, 4, 5) ceramic block is tetragonal, which corroborates the XRD results of the ceramic block. Because the long-range polarization of BST-*x* mol% MgO ceramics was destroyed after adding MgO, the crystal structure changed.

### 3.6. Microstructure Analysis of BST-MgO Ceramic Block

**Figure 6:**
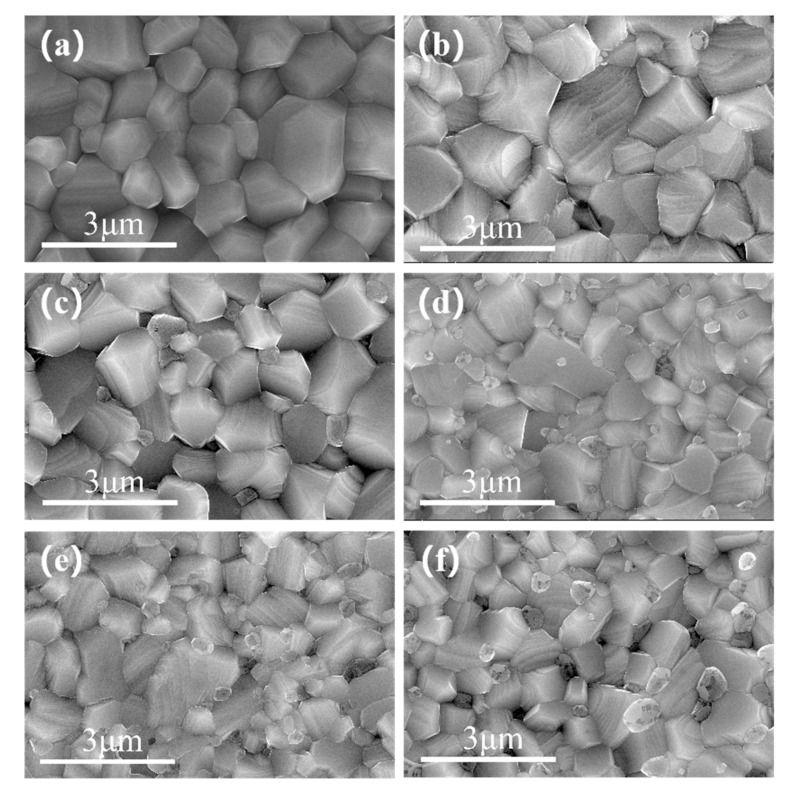
SEM images of the BST and BST-*x* mol% MgO (*x* = 1, 2, 3, 4, 5) ceramics: (**a**) BST, (**b**) *x* = 1, (**c**) *x* = 2, (**d**) *x* = 3, (**e**) *x* = 4, and (**f**) *x* = 5.

**Figure 7:**
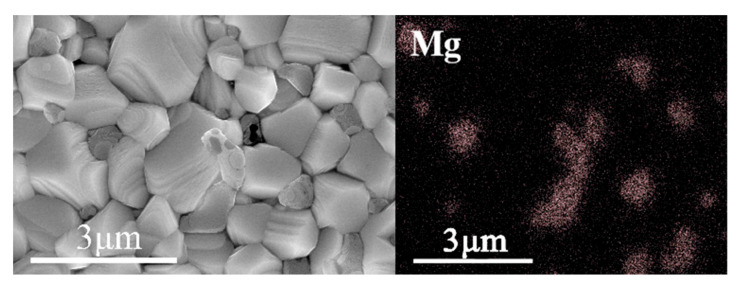
EDS maps of the BST-2 mol% MgO ceramic.

The grain size of BST-x mol% MgO (*x* = 1, 2, 3, 4, 5) ceramics was approximately 0.5–1.5 μm, which is smaller than 1–2 μm grain size of BST ceramic (Figure 6). The grain size was evidently reduced after the addition of MgO. Figure 6b–f reveal the presence of heterogeneous particles at the grain boundary, and these particles increased with an increase in the MgO content. To check whether the heterogeneous particles were MgO, BST-2 mol% MgO ceramic blocks were scanned and analyzed by EDS (Figure 7). It can be seen from the figure that the dark particles had a high Mg content. Hence, the result confirmed that the heterogeneous particles were MgO and the gray particles were BST, thus further confirming the presence of MgO. With an increase in the MgO content, the grain size became smaller and smaller. Hence, MgO may act as a growth inhibitor and is often present at grain boundaries due to its low diffusion rate [21] as foreign ions preferentially accumulate at the grain boundaries. With an increase in MgO content, Mg^2+^ accumulated at the grain boundaries, thus hindering the further movement of grain boundaries and the growth of grains. In Figure 6, it also can be seen that there is a small amount of pores in the ceramic block, and the relative densities of the BST and BST-5 mol% MgO ceramic blocks are calculated by Archimedes’ principle to be 94% and 97.8%, respectively. This indicates that the MgO particles fill the pores of the ceramic bulk and improve the density of the BST-x mol% MgO ceramic bulk.

### 3.7. Dielectric Properties of BST-MgO Ceramics at Room Temperature

**Figure 8:**
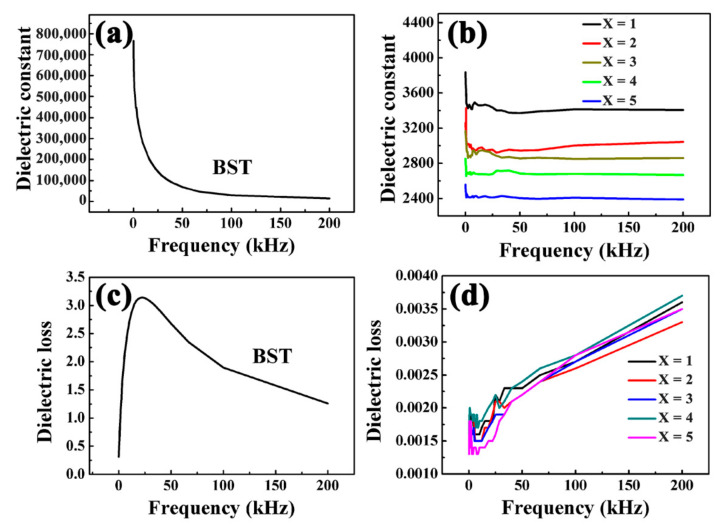
Dielectric constant-frequency curves of the (**a**) BST and (**b**) BST-*x* mol% MgO (*x* = 1, 2, 3, 4, 5) ceramics. Dielectric loss-frequency curves of the (**c**) BST and (**d**) BST-*x* mol% MgO (*x* = 1, 2, 3, 4, 5) ceramics.

Figure 8 reveals that at low frequencies and room temperature, the dielectric constant of BST ceramics reached 760,000, and then gradually decreased with an increase in frequency. The dielectric constant decreased to around 13700 when the frequency was greater than 200 kHz.

As seen in Figure 8b, when BST ceramics are doped with MgO, the dielectric constants of the ceramic bulk decrease significantly. The dielectric constants of BST-x mol% MgO are 3850(*x* = 1), 3400(*x* = 2), 3200(*x* = 3), 2800(*x* = 4), 2600(*x* = 5) at a frequency of 0.12 kHz. As the frequency exceeds 2 kHz, the dielectric constant gradually stabilizes. The dielectric constants of BST-x mol% MgO were stable at approximately 3400(*x* = 1), 3000(*x* = 2), 2900(*x* = 3), 2700(*x* = 4), 2400(*x* = 5), respectively. The dielectric constant of BST-1 mol% MgO has a maximum value, which is 1/3 [24] times higher than that of ordinary BT-MgO composite ceramics. The key factors that led to a change in the dielectric properties of BT ceramics were the doping effect, size effect, and oxygen vacancy concentration. The decrease in the dielectric constants of BST-*x* mol% MgO ceramics at room temperature was due to the transformation of the BST-*x* mol% MgO ceramic structures from a pseudo cubic structure to tetragonal structure due to MgO doping [26]. Simultaneously, the grain size of BST-*x* mol% MgO ceramics decreased evidently. A very small grain size will reduce the dielectric constant [27]. Although the addition of MgO improves the compactness of BST-x mol% MgO ceramics, due to the low dielectric constant of MgO and the aggregation at the grain boundaries of BST ceramics, it affects the polarization of BST-MgO ceramics and ultimately affects the dielectric properties of MgO ceramic bulk [21]. The dielectric loss of BST initially increased and then decreased with an increase in frequency by Figure 8c. When the frequency was 25 kHz, the maximum dielectric loss was approximately 3. Figure 8d also shows that the dielectric losses of BST-*x* mol% MgO (*x* = 1, 2, 3, 4, 5) ceramics and BST were both evidently reduced to less than 0.004. This dielectric loss was equivalent to 2/3 [24] that of BaTiO_3_-MgO composite ceramics. With the increase in MgO doping amount, the dielectric loss of BST-MgO ceramics decreased gradually. The reason for the decrease in dielectric loss may be due to the change in the structure of BST-MgO ceramics and the decrease in polarization. Furthermore the aggregation of MgO at the grain boundaries destroys the long-range polarization of BST ceramics.

### 3.8. Analysis of Dielectric Properties of BST-MgO Ceramics at Variable Temperatures

**Figure 9:**
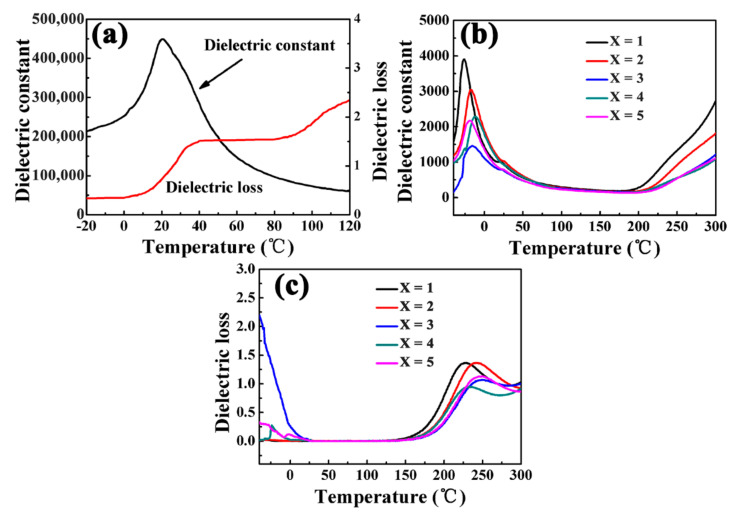
Dielectric constant-temperature curves of the (**a**) BST and (**b**) BST-*x* mol% MgO *(x* = 1, 2, 3, 4, 5) ceramics. (**c**) Dielectric loss-temperature curves of BST-*x* mol% MgO (*x* = 1, 2, 3, 4, 5) ceramics.

Figure 9 shows the temperature-variable dielectric properties of BST and BST-x mol% MgO ceramic bulk at 1 kHz. The temperature ranges tested were −20 °C to 120 °C (BST) and −65 °C to 300 °C (BST-x mol% MgO). Figure 9a shows that the dielectric constant of BST ceramics was the highest at 20 °C with a value of 450,000. With an increase in temperature, the dielectric constant drastically decreased but still reached 40,000. From Figure 9a,b, it can be found that the temperature-dependent permittivity of BST-x mol% MgO was significantly lower than that of BST ceramics. The dielectric constants at Curie temperature of BST-x mol% MgO ceramics were 3980(*x* = 1), 3050(*x* = 2), 2300(*x* = 3), 2200(*x* = 4), and 1450(*x* = 5), respectively. The Curie temperature of BST ceramics is 20 °C, while the Curie peak of BST-x mol% MgO was below 0 °C. The substitution of Ti^4+^ by Mg^2+^ resulted in the formation of oxygen vacancies, while the Curie temperature shift was mainly due to the accumulated oxygen vacancies [28]. Figure 9c shows that the dielectric loss at variable temperatures of BST-*x* mol% MgO was also obviously lower than that of BST.

## 4. Conclusions

In this study, BST and BST-MgO ceramics were prepared. The results revealed that the addition of MgO to the BST matrix can significantly inhibit grain growth and improve its compactness, which was consistent with the reported results for other BT-MgO systems. The addition of MgO provided excellent dielectric properties for materials. The dielectric constant of BST-1 mol% MgO was 3850 at room temperature, and the dielectric loss of BST-*x* mol% MgO was less than 0.004. Its dielectric constant was 1/3 higher than that of an ordinary BT-MgO composite ceramic. The dielectric loss was equivalent to 2/3 that of BT-MgO composite ceramics. The addition of MgO significantly improved the dielectric properties of the material and caused the Curie peak to shift toward lower temperature. The dielectric properties of the BT-MgO system may be further improved by changing the doping elements or adjusting the doping ratio. These new findings help in improving the dielectric properties of the BT-MgO system and provide valuable information for research implied in capacitors functioning at a low temperature.

## Figures and Tables

**Table 1:** Lattice parameters of the BST and BST-*x* mol% MgO (*x* = 1, 2, 3, 4, 5) powders.

Sample	*a*-Axis (Å)	*c*-Axis(Å)	*c*/*a*
BST	3.9883	3.9838	0.9989
*x* = 1	3.9884	3.9884	1
*x* = 2	3.9889	3.9886	0.9999
*x* = 3	3.9887	3.9985	1.0024
*x* = 4	3.9893	3.9887	0.9998
*x* = 5	3.9888	3.9888	1

**Table 2:** Lattice parameters of the BST and BST-*x* mol% MgO (*x* = 1, 2, 3, 4, 5) ceramics.

Sample	*a*-Axis (Å)	*c*-Axis(Å)	*c*/*a*
BST	3.9808	3.9899	1.0020
*x* = 1	3.9884	3.9984	1.0025
*x* = 2	3.9881	3.9996	1.0028
*x* = 3	3.9872	3.9995	1.0030
*x* = 4	3.9863	3.9990	1.0031
*x* = 5	3.9860	3.9998	1.0035

## Data Availability

No new data were created or analyzed in this study. Data sharing is not applicable to this article.
